# Performance study of ZnO-TPU/CS bilayer composite electrospinning scaffold in skin wound healing

**DOI:** 10.3389/fbioe.2025.1636932

**Published:** 2025-09-17

**Authors:** Jinlong Wang, Guoxing Huang, Quan Qin, Nianhua Dan, Xinlou Li, Kai Sun, Yuan Yang, Meng Wang

**Affiliations:** ^1^ 306th Clinical College of PLA, The Fifth Clinical College, Anhui Medical University, Beijing, China; ^2^ Department of Orthopaedics, The Ninth Medical Center of Chinese PLA General Hospital, Beijing, China; ^3^ Key Laboratory of Leather Chemistry and Engineering of Ministry of Education, Sichuan University, Chengdu, China; ^4^ Research Center of Biomedical Engineering, Sichuan University, Chengdu, Sichuan, China; ^5^ Department of Medical Research, Key Laboratory of Environmental Sense Organ Stress and Health of the Ministry of Environmental Protection, The Ninth Medical Center of Chinese PLA General Hospital, Beijing, China; ^6^ PLA Rocket Force Characteristic Medical Center, Beijing, China; ^7^ Department of Aerospace Clinical Medicine, The Ninth Medical Center of Chinese PLA General Hospital, Beijing, China

**Keywords:** electrospinning, skin wound healing, zinc oxide nanoparticles, biocompatibility, tissue engineering scaffold (TES)

## Abstract

**Introduction:**

The high incidence of skin injuries and the limitations of conventional dressings highlight the need for advanced wound care materials. Electrospun nanofibrous scaffolds, with their extracellular matrix-like architecture, offer potential to enhance healing.

**Methods:**

A bilayer nanofibrous scaffold of thermoplastic polyurethane (TPU) and chitosan loaded with zinc oxide nanoparticles (ZnO) (TPU/CS@ZnO) was fabricated via electrospinning. The scaffold consisted of a hydrophobic TPU outer layer for waterproof protection and a hydrophilic TPU/CS@ZnO inner layer for bioactivity. Physicochemical properties were characterized by morphology, mechanical strength, and wettability. Cytocompatibility was evaluated in vitro, and wound healing efficacy was tested in vivo using a full-thickness skin defect model.

**Results:**

The scaffold displayed uniform fibres with a base-layer diameter of 231.81 ± 44.85 nm, tensile strength of 8.42 ± 0.58 MPa, and Young’s modulus of 17.96 ± 0.78 MPa. Water contact angles confirmed hydrophilic and hydrophobic layer characteristics (52.68° ± 4.46° vs. 113.60° ± 2.85°). In vitro studies showed enhanced cell proliferation and adhesion, while in vivo experiments demonstrated over 90% wound closure at day 14, significantly faster than untreated groups. Histological analysis indicated contributions from cellular adhesion, angiogenesis, and immunomodulation.

**Discussion:**

The bilayer TPU/CS@ZnO scaffold integrates structural protection with biological activity, accelerating wound repair through multiple mechanisms. These findings support its potential as a multifunctional wound dressing, while further studies are needed to clarify molecular pathways and advance clinical application.

## Graphical Abstract

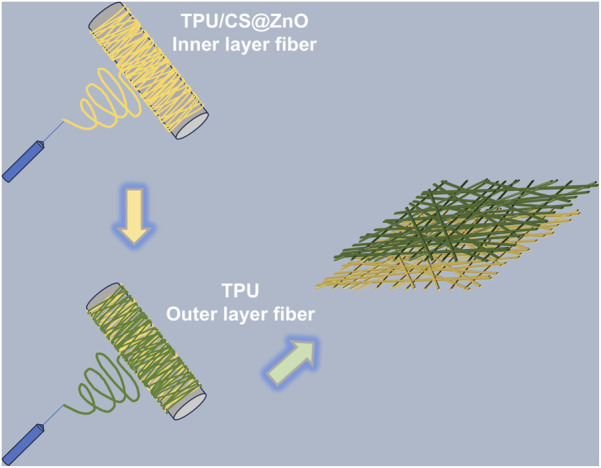

## 1 Introduction

The skin, as the largest organ of the human body, not only serves as a physical barrier against the invasion of external pathogens but also plays a pivotal role in thermoregulation, sensory perception, and the maintenance of internal homeostasis (Rognoni and Watt, 2018). Among all types of traumas, skin injuries occur with the highest frequency. The primary causes of skin damage include mechanical trauma (such as abrasions, lacerations, penetrating wounds or surgical incisions), as well as exposure to radiation, electricity, corrosive chemicals or thermal sources (Gurtner et al., 2008). When skin damage exceeds a certain threshold, it can result in the loss of normal function and may be accompanied by severe infection (Luo et al., 2019). Therefore, promoting wound repair and improving the quality of healing hold significant clinical value in reducing both disability and mortality (Liu et al., 2025). Key factors that contribute to accelerated wound healing include haemostasis, debridement, protection against foreign bodies and pathogens, prevention or treatment of infection, maintenance of a moist environment to avoid wound desiccation, management of wound exudate, stimulation of keratinocyte and fibroblast proliferation and migration, enhancement of re-epithelialisation, as well as the promotion of angiogenesis and connective tissue formation (Clark, 2001; Naseraei et al., 2025). The application of dressings to promote wound healing is a well-established and effective therapeutic approach. However, traditional wound dressings such as gauze merely serve to cover the wound surface, lacking inherent antibacterial or pro-healing properties. Moreover, they are prone to adhering to the wound bed, potentially causing secondary injury and increasing the risk of infection. As a result, these conventional materials no longer meet the evolving demands of modern wound care.

Electrospinning is an efficient technique for the fabrication of nanoscale biomaterials (Garkal et al., 2021). Owing to its simplicity, cost-effectiveness, and versatility, it has been widely employed across diverse fields, including biomedicine, food packaging, and environmental protection (Currie et al., 2020). The fibrous scaffolds produced by electrospinning are characterised by high porosity and mechanical strength, with fibre diameters typically ranging from 100 to 1,000 nm (Kulkarni et al., 2025). These properties enable them to closely mimic the structure of the extracellular matrix, thereby providing a favourable microenvironment for cellular adhesion, proliferation, and differentiation (Agarwal et al., 2009). Moreover, electrospun nanofibres serve as excellent drug delivery systems (Kulkarni et al., 2022). Their unique structural features and tunable fabrication parameters allow for the incorporation of a wide range of therapeutic agents, including antibiotics, bioactive molecules, and metal nanoparticles (Rieger et al., 2013). Additionally, by modulating fibre architecture and composition, it is possible to achieve controlled drug release profiles, such as initial burst release, sustained release or stimuli-responsive release, tailored to specific therapeutic needs (Lai et al., 2014).

Thermoplastic polyurethane (TPU), a cost-effective and easily processed synthetic material, has attracted considerable attention due to its excellent mechanical properties, structural tunability, and favorable biological performance. With high tissue compatibility and low immunogenicity, TPU has been FDA-approved for medical use in tissue engineering applications (Nakhaei et al., 2023). Chitosan (CS), a natural biopolymer, also holds great promise in the biomedical field. It exhibits excellent biocompatibility, inherent antibacterial properties, and biodegradability. These advantageous characteristics, along with its diverse range of derivatives, have made chitosan widely applicable across various domains of biomedicine (Asghari et al., 2022). At present, chitosan is widely utilised in a variety of material forms, including haemostatic sponges, hydrogels, electrospun scaffolds, dry films, and collagen-based composites (Yang et al., 2025; Xiong et al., 2025).

Zinc is an essential element for human growth and development, predominantly existing in the body in its ionic form, mainly within the cytoplasm and extracellular fluid. It plays a critical role in regulating enzyme activity, signal transduction, and gene expression. Zinc ions can also bind to macromolecules such as proteins and nucleic acids, contributing to structural stability, catalysis, and transcriptional regulation. Moreover, zinc is involved in a range of biological processes, including antioxidation and immune modulation (Dodero et al., 2020). In recent years, nanoscale zinc oxide (ZnO) particles have garnered significant research interest. Incorporating metal nanoparticles into fibrous materials can alter their crystalline phase and enhance their mechanical properties. Furthermore, ZnO nanoparticles can serve therapeutic functions. Upon release into the body, ZnO can generate reactive oxygen species (ROS), conferring broad-spectrum antimicrobial activity. In parallel, zinc ions, as essential micronutrients, promote cellular proliferation and tissue regeneration, thereby contributing to the wound healing process (Sasan et al., 2024).

Polyurethane-chitosan-based nanofibres have gained widespread application in various biomedical implants due to their excellent tissue compatibility. These composites demonstrate significant potential for coronary disease treatment (Ahmadi et al., 2021), neural regeneration (Huang et al., 2011), and vascular repair (Selvaras et al., 2023). However, hybrid nanofibres combining these two materials remain unexplored for wound healing applications. Furthermore, multilayer biomimetic scaffolds address the functional limitations of single-layer electrospun matrices by integrating strata with distinct functionalities, enabling the development of multifunctional biomaterials. While Marjan Mirhaj et al. fabricated an antimicrobial multilayer scaffold for skin regeneration via 3D printing and electrospinning (Mirhaj et al., 2023), the reliance on 3D printing introduces manufacturing complexity and elevated production costs that may limit clinical translation in wound management. Similarly, Serdar Tort et al. developed a trilayer nanofibrous dressing (Tort et al., 2017), yet its design overlooks the dynamic requirements of wound microenvironments and incorporates coaxial electrospinning—a process whose complexity may restrict practical scalability.

Polyurethane-chitosan electrospun fibres, already utilized in diverse biomedical applications through functional substance loading, were innovatively engineered with zinc oxide nanoparticles in this study. When applied as wound dressings, these composite fibres demonstrated superior biological performance. Leveraging established multilayer composite technology, we constructed a multifunctional polymeric fibrous dressing. Compared to existing alternatives, this design offers significant advantages in cost-effectiveness and streamlined fabrication—attributes that position it as a highly promising solution for clinical wound management.

## 2 Experimental

### 2.1 Materials and chemicals

TPU (Tecoflex™ SG-80A) was supplied by Lubrizol Corporation, Germany. Chitosan (Mn = 200,000–300,000) and zinc oxide nanoparticles (50 ± 10 nm) were purchased from Aladdin Biochemical Technology Co., Ltd. (Shanghai, China). The solvents, hexafluoroisopropanol (HFIP, purity 99.9%) and acetic acid (purity 99%), were obtained from Macklin Biochemical Technology Co., Ltd. (Shanghai, China). All other chemicals used in this study were of analytical grade, unless otherwise specified.

### 2.2 Preparation of electrospun TPU/CS@ZnO nanofibrous scaffolds

TPU was dissolved in HFIP to prepare an 8% (w/v) TPU solution. CS was dissolved in a 90% aqueous acetic acid solution to yield a 3% (w/v) CS solution. A predetermined amount of ZnO nanoparticles was added to the TPU solution and ultrasonicated for 2 h to ensure homogeneous dispersion of the nanoparticles. The TPU and CS solutions were then mixed thoroughly at a volumetric ratio of 80:20.

The bilayer scaffold was fabricated using an electrospinning apparatus (ET-M1, Beijing Ucalery Technology and Development Co., Ltd., China), a specialized device for generating nanoscale fibres. The bilayer scaffold was fabricated via a sequential two-step electrospinning process: Firstly, Co-electrospinning of polyurethane-chitosan-ZnONPs composite solution. Secondly, Direct electrospinning of pure polyurethane solution onto the TPU-CS-ZnONPs hybrid fibre mat. The resulting bilayer nanofibrous scaffold was then peeled off the aluminium foil collector and vacuum-dried at 40 °C for 24 h.

This homogeneous spinning solution was loaded into a syringe and electrospun at a voltage of 17 kV. The syringe needle was connected to the positive electrode, while the collector—an aluminium foil-covered rotating drum—was connected to the negative electrode. The distance between the needle and the collector was set at 10 cm, and the solution flow rate was maintained at 1.5 mL/h. Electrospinning was carried out for 9 h to form the initial nanofibrous scaffold.

Subsequently, TPU was dissolved in a mixed solvent of N, N-dimethylformamide (DMF) and acetone (75:25, v/v) to obtain an 18% (w/v) TPU solution. This solution was transferred to a syringe and electrospun at 20 kV, with a needle-to-collector distance of 20 cm and a flow rate of 0.8 mL/h. The spinning was continued directly onto the previously fabricated scaffold for an additional 6 h to form the complete bilayer structure. All scaffolds were then dried in a forced-air oven for 24 h to remove residual solvents.

### 2.3 Physical characterisation

#### 2.3.1 Morphology and elemental composition

The morphology and fibre dimensions of the nanofibrous scaffolds were examined using a scanning electron microscope (SEM, Apreo 2S, Thermofisher, United States). First, samples were trimmed to an appropriate size and mounted onto the sample holder using conductive adhesive tape. Subsequently, the sample surface was coated with a 4-nm-thick gold (Au) layer. SEM imaging was then performed at an accelerating voltage of 8 kV. Finally, 50 fibres were randomly selected from the obtained images. Their diameters were measured to generate a fibre diameter distribution plot. In addition, the elemental composition and spatial distribution of elements within the samples were analysed using energy-dispersive spectroscopy (EDS).

#### 2.3.2 Mechanical properties

The tensile strength and elongation at break of the scaffolds were assessed using a universal testing machine (GT AI 7000S, gotech, China). Samples were cut into dumbbell-shaped strips with a working area of 40 mm × 10 mm. The widened ends of the dumbbell-shaped specimens were clamped securely, ensuring the working section was fully extended but not under tension. The stretching speed was set at 10 mm/min, and each sample was pulled until rupture.

#### 2.3.3 Water contact angle

The hydrophilicity of the scaffolds was evaluated using a contact angle goniometer (DSA-X, betops Co., Ltd., China). Samples were cut into 10 mm × 10 mm squares and mounted flat onto standard glass slides. A droplet of ultrapure water (3–5 μL) was vertically dispensed onto the scaffold surface. The state of the droplet on the scaffold surface was recorded using a high-speed camera. After the droplet reached equilibrium, an image was immediately captured. The contact angle of the scaffold was determined by analyzing the tangent line at the droplet-scaffold interface using ImageJ software.

#### 2.3.4 Air permeability

The air permeability of scaffolds was evaluated using a digital air permeability tester (YG461E, Meibangyiqi, China). Test fabrics were conditioned in a standard atmosphere (GB/T 6529: 20 °C ± 2 °C, 65% ± 4% RH) for at least 24 h prior to testing. Specimens were then smoothly positioned over the lower clamp ring of the test head, ensuring a wrinkle-free state without localized slackness or tension. The upper clamp ring was lowered and uniformly tightened to secure the sample. A differential pressure (ΔP) of 100 Pa was set, and the system automatically recorded the steady-state airflow rate (Q).

#### 2.3.5 Water vapor transmission rate (WVTR)

Freshly distilled water was injected into permeation cups to a depth of 19 mm below the specimen surface. Specimens were cut to fully cover cup orifices and secured airtight using sealing wax. Sealed assemblies were equilibrated for 1 h in a climate chamber (YG601H-Ⅱ nbdahe China). Controlled environment: 38 °C ± 0.5 °C, 50% ± 5% RH. Initial mass (W_0_) recorded post-equilibration. Subsequent mass measurements (W_1_–W_6_) taken at 8-h intervals over 48 h. Cumulative mass loss vs. time plotted to verify linearity (*R* ^2^ > 0.98 required).

$$\text{WVT }rate=\frac{W_{0}-W_{6}}{A\times t}$$

A: Effective test area (m^2^) (inner orifice cross-section), t: Total test duration (48 h).

### 2.4 Cytocompatibility

A haemolysis assay was conducted to assess the haemocompatibility of the scaffolds. Red blood cells were collected from rats and adjusted to a concentration of 4%. An equal volume of scaffold extract was added to the cell suspension and incubated at 37 °C for 3 h. After incubation, the mixture was centrifuged at 2,000 rpm for 10 min, and the absorbance of the supernatant was measured at 545 nm. The haemolysis rate was calculated accordingly. A 2% Triton X-100 solution was used as the positive control (complete haemolysis), and physiological saline served as the negative control.

Cytotoxicity was evaluated using the Cell Counting Kit-8 assay (CCK-8, CK04, Dojindo Japan). Murine fibroblasts (L929 cells, Procell Life Science & Technology Co., Ltd., China) were seeded in 96-well plates at a density of 1 × 10^4^ cells per well. Each well was treated with 100 μL of scaffold extract and incubated for 1, 3, 5, and 7 days. Cell viability was determined using the CCK-8 colourimetric assay, and the absorbance was measured at 450 nm using a microplate reader (Synergy, Invitrogen, United States) to calculate the relative cell viability.

### 2.5 Assessment of cell proliferation and invasion

#### 2.5.1 Cell proliferation assay

To assess cellular proliferation, the nanofibrous dressings were placed at the bottom of 24-well plates. L929 fibroblasts were seeded at a density of 5 × 10^4^ cells/mL, with 200 μL of cell suspension added per well. The plates were incubated at 37 °C in a 5% CO_2_ atmosphere for 3 days. Following incubation, a live/dead cell staining kit was applied (C2017S Shanghai Beyotime Biotechnology Co., Ltd., China), and the samples were incubated for 30 min in the dark at 37 °C. Fluorescence microscopy was used to visualise and assess cell viability.

#### 2.5.2 Scratch (wound healing) assay

Cells were seeded in 6-well plates at a density of 1 × 10^5^ cells per well. Once the cells reached approximately 80% confluency, a sterile pipette tip was used to create a linear scratch in the monolayer. Detached cells were removed. The culture medium was then replaced with sample extract, followed by 24-h incubation in a cell culture incubator. Bright-field images of the scratch regions were captured at 0 h and 24 h post-scratch creation under a phase-contrast microscope. (IX81, OLYMPUS, Japan). The scratch closure area was quantified using ImageJ software to calculate the percentage of wound healing, thereby evaluating the scaffold’s effect on cell migration.

$$Wound\;healing\;percentage\left(\%\right)=\frac{{area}_{0h}-{area}_{24h}}{{area}_{0h}}\times 100\%$$

Area_0h_ denotes the initial wound area at the time of wound creation, while area_24h_ represents the wound area after 24 h of cell treated with the sample extracts.

#### 2.5.3 Transwell migration and invasion assay

Cells were seeded in the upper chamber of a Transwell insert (with a porous membrane) at a density of 5 × 10^4^ cells per well. The lower chamber was lined with fibrous scaffolds. After 24 h of incubation at 37 °C in a 5% CO_2_ environment, non-migrated cells on the upper surface of the membrane were removed. The migrated or invaded cells on the lower surface were fixed with methanol and stained with crystal violet. Migrated cells were observed and counted under a microscope. The number of migrating/invading cells was quantified to compare differences between experimental groups.

### 2.6 *In vivo* evaluation of wound healing

A total of 18 male Sprague-Dawley (SD) rats, aged 8 weeks and weighing between 250 and 300 g, were selected to evaluate the *in vivo* wound healing efficacy of the nanofibrous scaffolds. The animals were anaesthetised with an intraperitoneal injection of 3% sodium pentobarbital solution. Two full-thickness circular skin wounds, each 15 mm in diameter, were surgically created on either side of the spinal midline on the dorsum of each rat. The wound on the right side served as the control group and was covered with Petrolatum Gauze (Sterile, Fine Mesh, Zhende Medical Co., Ltd., China), while the wound on the left side received treatment with the nanofibrous scaffold (experimental group). Digital photographs of the wound sites were taken on days 7, 14, and 21 post surgery to monitor the healing process. At each time point, six rats were randomly euthanised, and the wound tissues were harvested and fixed in 4% paraformaldehyde for subsequent histological analyses. The wound area was quantified using ImageJ software based on the digital images obtained.

### 2.7 Histological analysis

The harvested skin tissues were fixed, dehydrated, and cleared before being embedded in paraffin to form tissue blocks. These blocks were sectioned into 5 μm-thick slices. Haematoxylin and eosin (H&E) staining and Masson’s trichrome staining were then performed on the sections. Following staining, the samples were dehydrated, cleared, and mounted using a neutral resin. Histological images were acquired using a whole-slide scanner.

For immunohistochemical analysis, antigen retrieval was first performed, followed by quenching of endogenous peroxidase activity and blocking of non-specific binding. Samples were sequentially incubated with primary antibodies against VEGF (ab317030, Abcam, United States), bFGF (ab321990, Abcam, United States), and CD68 (ab283654, Abcam, United States), followed by secondary antibody incubation and Streptavidin-Biotin Complex (SABC) treatment. After colour development and nuclear counterstaining, the sections were dehydrated, cleared, and mounted. A slide scanner was used to visualise and analyse the spatial distribution of target protein expression.

### 2.8 Statistical analysis

All data are presented as the mean ± SD. Bilateral t-test was used to compare differences between groups. Statistical significance was indicated as follows: *p < 0.05; **p < 0.01; ***p < 0.001.

## 3 Results

### 3.1 Morphology of the fibre scaffold and cytotoxicity screening

**FIGURE 1:**
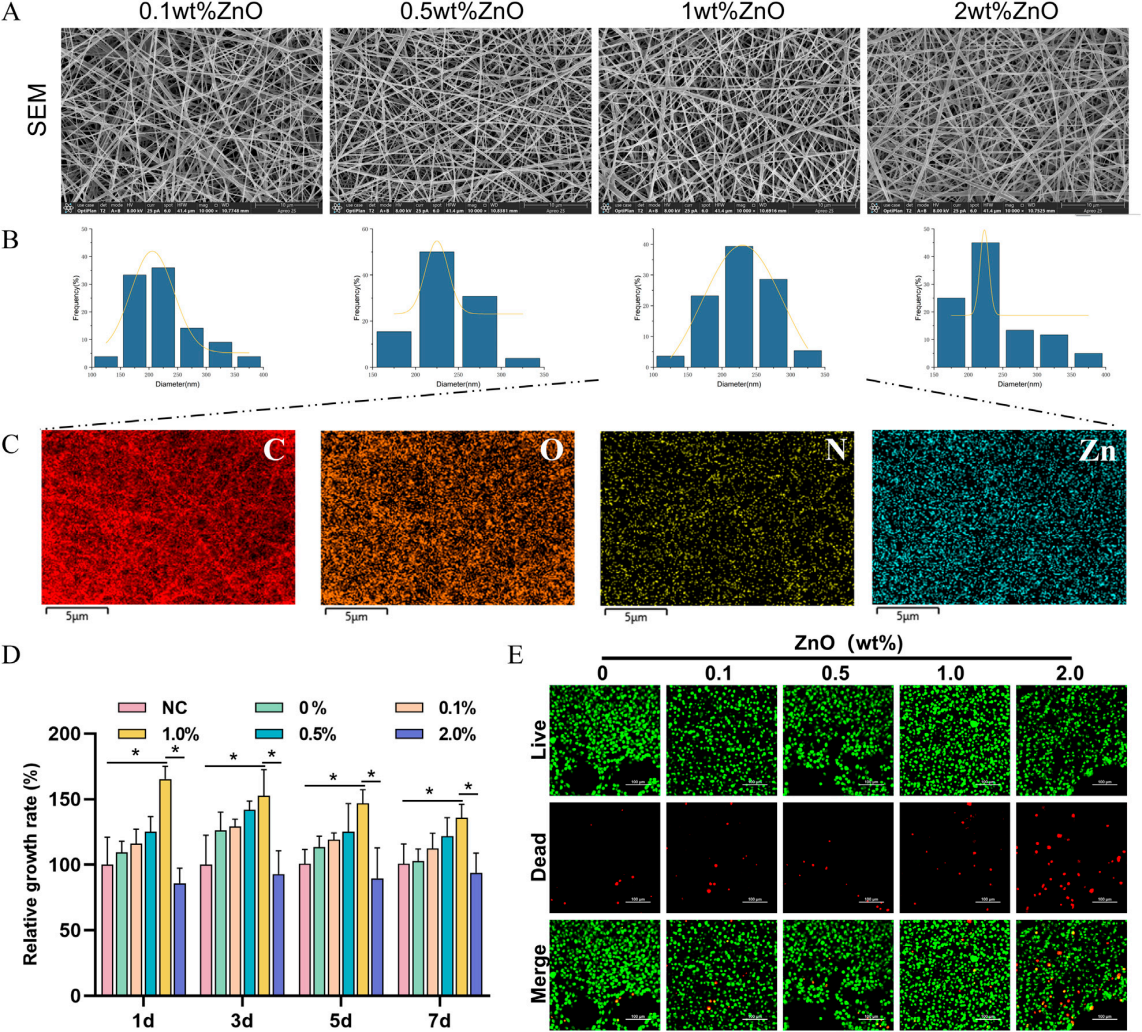
Screening of ZnO nanoparticle loading amounts. **(A)** SEM images of polyurethane/chitosan composite fibres loaded with ZnO nanoparticles at varying concentrations (ZnO contents of 0.1 wt%, 0.5 wt%, 1.0 wt%, and 2.0 wt%). Scale bar: 30 μm. **(B)** Histogram of nanofibre diameter frequency distribution. **(C)** Elemental mapping by EDS. Scale bar: 5 μm. **(D)** Cytotoxicity of scaffold extracts from each group evaluated using the CCK-8 assay. **(E)** Assessment of cell viability after 3 days of culture on each scaffold group using live/dead staining. Scale bar: 50 μm. All cell-based experiments were conducted using L929 fibroblasts. All data are presented as mean ± SD (*n* ≥ 3). Statistical analysis was performed using a two-tailed t-test; *P < 0.05, **P < 0.01, ***P < 0.001.

We successfully fabricated TPU/CS electrospun nanofibre scaffolds loaded with ZnO nanoparticles using electrospinning technology. First, the morphology and fibre distribution of the scaffolds were observed using SEM. As shown in Figure 1A, the nanofibres were continuous and smooth, with a uniform distribution across the field of view. The fibre diameter in the observed area followed a normal distribution, with an average diameter of 231.81 ± 44.85 nm (Figure 1B), indicating stable electrospinning parameters and good material compatibility. Elemental analysis of the fibre scaffold was performed using energy-dispersive X-ray spectroscopy (EDS). The results revealed that the Zn signal was evenly distributed throughout the scaffold, while the EDS analysis also showed the distribution and proportions of C, O, and N elements (Figure 1C). In summary, ZnO nanoparticles were uniformly incorporated into the TPU/CS nanofibres.

Given the potential cytotoxic effects of ZnO, excessive amounts may inhibit cell proliferation. Therefore, we employed the CCK-8 assay to assess the relative cell proliferation and determine an optimal ZnO loading concentration. As shown in Figure 1D, the presence of lower concentrations of ZnO at time points 1, 3, 5, and 7 days promoted cell proliferation. However, 2% ZnO exerted an inhibitory effect on cell proliferation. Cell viability and death staining revealed that 2% ZnO led to a significant increase in the number of dead cells, while the number of dead cells in the low-concentration ZnO groups was almost identical to that of the control group (Figure 1E). Based on these results, the 0.1% and 0.5% ZnO concentrations showed limited effects on cell proliferation, while 2% ZnO exhibited cytotoxicity. These three groups were not further investigated. In subsequent studies, TPU/CS@ZnO refers to the scaffold with a 1.0% ZnO loading.

### 3.2 Physical properties characterisation of the fibre scaffold

**FIGURE 2:**
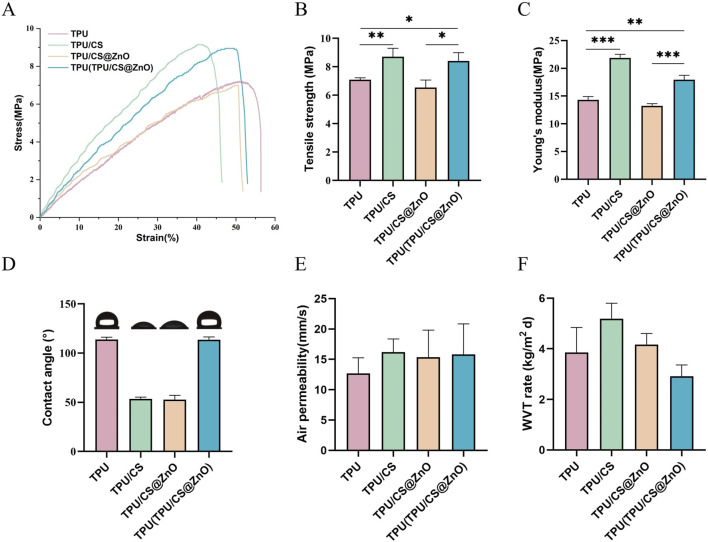
Physical properties of TPU, TPU/CS, TPU/CS@ZnO, and layered TPU(TPU/CS@ZnO) Scaffolds. **(A–C)** Stress–strain curves, tensile strength, and Young’s modulus of the various scaffold groups to evaluate mechanical performance. **(D)** Surface wettability analysis. **(E)** Air permeability assessment. **(F)** WVTR of scaffolds. All data are presented as mean ± SD (*n* ≥ 3). Statistical comparisons were conducted using two-tailed t-tests; *P < 0.05, **P < 0.01, ***P < 0.001.

Wound dressings require certain tear resistance and high extensibility to provide adequate support for cell proliferation and tissue remodelling. We measured the breaking elongation and tear strength of the fibre scaffolds using a universal testing machine. The results showed that the TPU-based fibre scaffolds exhibited good extensibility and mechanical properties. Although the incorporation of CS and ZnO nanoparticles slightly reduced the extensibility and strength of the scaffolds, the composite bilayer scaffold structure effectively compensated for these drawbacks (Figures 2A–C).

We also evaluated the hydrophilic properties of the fibre scaffolds with different components using a contact angle goniometer. The results indicated that the water contact angle of the TPU/CS and TPU/CS@ZnO groups was approximately 50°, indicating hydrophilic properties, with no significant difference between the two groups. The water contact angle of the TPU layer fibre scaffolds was greater than 90°, demonstrating excellent hydrophobic properties (Figure 2D).

Air permeability is another important characteristic for wound dressings. Using a fabric permeability tester, we measured the air permeability of the scaffolds. The results showed that all scaffold groups exhibited excellent air permeability, which ensures a favourable environment for wound healing (Figure 2E). The water vapour transmission properties of each scaffold group were also assessed, with the results presented in Figure 2F. All scaffolds exhibited favourable water vapour permeability, indicating their potential to effectively maintain an optimal wound environment conducive to healing.

### 3.3 *In vitro* biocompatibility of the scaffold

**FIGURE 3:**
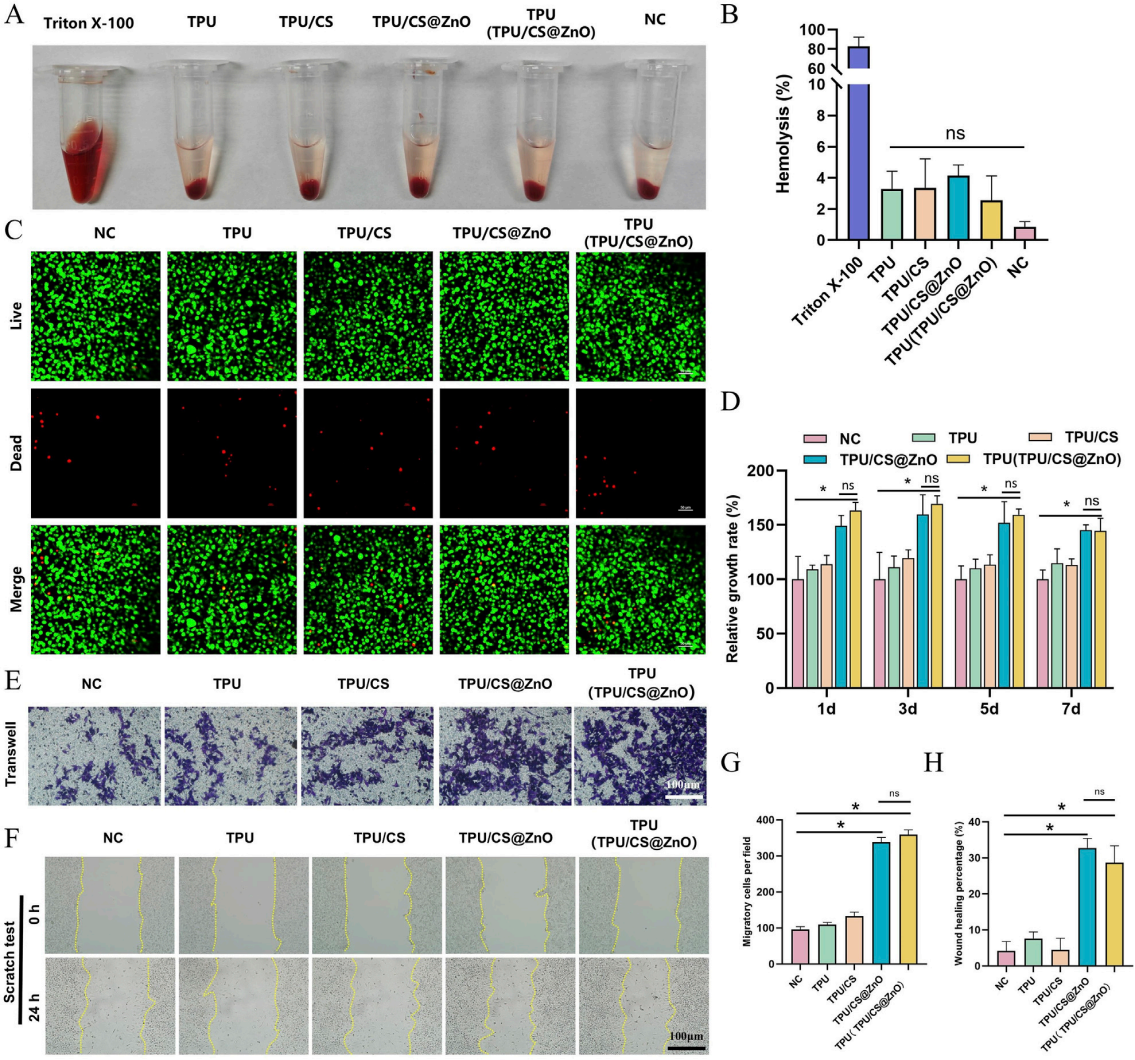
*In vitro* biocompatibility and functional evaluation of scaffolds. **(A)** Macroscopic images of haemolysis assays for TPU, TPU/CS, TPU/CS@ZnO, and TPU(TPU/CS@ZnO) scaffold groups. **(B)** Quantification of haemolysis rates. Triton X-100 served as a positive control; physiological saline was used as a negative control. Other groups were tested using scaffold extracts. **(C)** Live/dead staining of cells cultured on scaffolds for 3 days to assess cell viability. Scale bar: 50 μm. **(D)** Cell proliferation on the surface of each scaffold assessed by the CCK-8 assay. **(E)** Transwell assay to evaluate the impact of scaffolds on cellular invasion. Scale bar: 100 μm. **(F)** Scratch assay to assess cell migration. Scale bar: 100 μm. **(G)** Quantitative analysis of migrated cells from the transwell assay. **(H)** Quantification of wound closure area from the scratch assay. All cell experiments exclusively utilized the L929 fibroblast. All data are expressed as mean ± SD (*n* ≥ 3). Statistical analysis was conducted using a two-tailed t-test; ns indicates no statistical significance, *P < 0.05, **P < 0.01, ***P < 0.001.

Good biocompatibility is a necessary feature for wound dressings. Firstly, the haemolysis rate of the fibre scaffold extracts was determined. As shown in the figure, compared to the positive control group, all scaffold extracts, when mixed with blood cells, resulted in a clear supernatant, whereas the positive control group exhibited a bright red colour. The haemolysis rate was calculated based on the absorbance values of the supernatant, and all scaffold groups showed haemolysis rates of less than 5%, demonstrating good blood compatibility (Figures 3A,B).

To further assess tissue compatibility, we used mouse fibroblasts (L929 cells), which play a central role in wound healing. First, the survival of L929 cells on the scaffold surface was determined. Live/dead cell fluorescence staining showed only a few scattered red fluorescence signals, while the majority of cells displayed green fluorescence and exhibited a spindle-shaped normal cell morphology (Figure 3C). These results indicate that the TPU/CS@ZnO fibre scaffold possesses excellent biocompatibility and shows promise as a potential wound dressing.

### 3.4 Effect of the scaffold on fibroblast behavior

Wound dressings should promote cell proliferation and migration. We evaluated the biological performance of the scaffold in terms of these functions through *in vitro* experiments. First, to assess the scaffold’s ability to promote cell proliferation, we cultured L929 cells in scaffold extracts and measured the relative cell proliferation rate using the CCK-8 assay. The results showed that the scaffolds loaded with ZnO nanoparticles promoted cell proliferation effectively at the 1, 3, 5, and 7-day time points (Figure 3D).

Furthermore, we seeded cells onto the scaffold surface and performed a scratch assay to investigate the impact of the scaffold on cell migration. The results showed that the scratches in the nanofibre scaffold group were significantly reduced at various degrees, whereas there was almost no change in the control group, indicating that the fibre scaffolds enhanced cell migration. Notably, the scaffolds loaded with ZnO nanoparticles demonstrated a stronger cell migration-promoting effect compared to the TPU and TPU/CS groups (Figures 3E,G). To further validate this effect, we performed a Transwell migration assay. The lower chamber of the Transwell was filled with the fibre scaffolds, and L929 cells were seeded in the upper chamber. After incubation for a certain period, we observed a large number of spindle-shaped cells on the surface of the ZnO-loaded scaffolds. In contrast, only a few cells were present in the control, TPU, and TPU/CS groups (Figures 3F,H). These results confirm that the ZnO-loaded fibre scaffolds effectively promote both cell proliferation and migration.

### 3.5 *In vivo* wound healing performance of the scaffold

**FIGURE 4:**
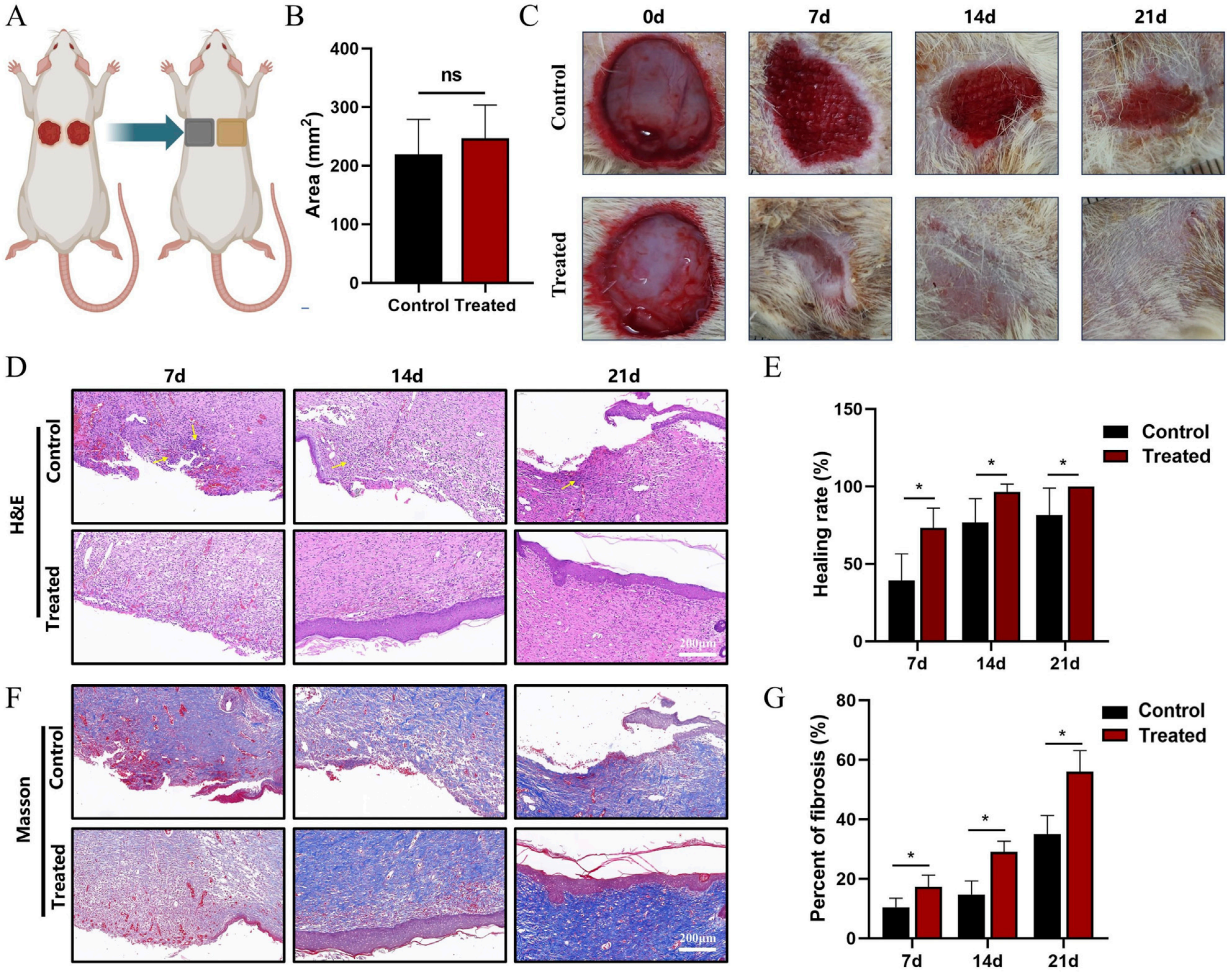
Evaluation of wound healing in a full-thickness skin injury animal model. **(A)** Schematic diagram of the animal model and grouping. The left and right flanks represent control and treatment groups, respectively. **(B)** Wound area measurement with paired t-test used for statistical analysis. **(C)** Macroscopic images of wounds on days 0, 7, 14, and 21. **(E)** Quantification of wound closure rates at days 7, 14, and 21. **(D)** H&E staining of wound tissues at days 7, 14, and 21. Scale bar: 200 μm. **(F)** Masson’s trichrome staining of wound tissues at corresponding time points. **(G)** Quantitative analysis of collagen fibre area as a proportion of total fibrous tissue. Scale bar: 200 μm. All data are presented as mean ± SD (*n* ≥ 6). Statistical significance determined via two-tailed t-test; ns indicates no significance, *P < 0.05, **P < 0.01, ***P < 0.001.

The above results suggest that TPU/CS@ZnO fibre scaffold has excellent potential as a wound dressing. To further assess its biological effects *in vivo*, we monitored the full-thickness skin wound healing process in rats. The study employed a self-control design to eliminate the impact of individual differences on wound healing (Figure 4A). Initially, the wound area on day 0 was measured. The results showed no statistically significant difference between the treatment and control groups, indicating that any differences in wound healing during subsequent treatment were unrelated to initial wound size (Figure 4B).

At the 7, 14, and 21-day time points, the healing rate in the treatment group was significantly higher than in the control group. On day 7, the wound healing rate in the treatment group had exceeded 70%, and by day 14, the wound was almost fully healed. In contrast, the control group still had some unhealed tissue even at day 21. During the healing process, newly formed granulation tissue almost grew into the control group’s gauze, causing adherence between the dressing and the tissue. This adhesion could damage the wound during dressing changes, leading to bleeding. However, the nanofibre scaffold in the treatment group showed minimal adherence to the wound, providing effective protection for the healing tissue (Figures 4C,E).

### 3.6 Histological staining analysis

Histological staining was used to assess the wound healing process at the cellular level, providing clear observations of granulation tissue filling, epidermal formation, and fibre remodelling. In H&E-stained images, the healing process in the control group showed a gradual filling of the wound area with newly formed granulation tissue, followed by the formation of a dense epidermal layer as remodelling occurred. A comparison of the two groups at the same time points revealed that the treatment group exhibited a significantly faster healing process. While the wound in the control group was not completely filled, the treatment group had already started to form epidermal tissue. By day 14, the wound area in the treatment group was almost fully repaired. On the other hand, in the later stages of healing, the control group still showed a significant presence of inflammatory cells, while the treatment group had almost no inflammatory cells remaining. This indicates that the treatment group exhibited superior wound healing, and that the fibre scaffold dressing also had an anti-inflammatory effect (Figure 4D).

Additionally, Masson’s trichrome staining was used to observe collagen fibre formation, distribution, and remodelling in the newly formed tissue. In the treatment group, collagen deposition in the wound area was more uniform, with higher staining intensity, indicating rich collagen synthesis and a more orderly arrangement. Particularly in the dermal layer, collagen fibres were densely distributed and aligned in a parallel fashion, suggesting better recovery of dermal structure during the healing process. Simultaneously, fibre formation occurred alongside angiogenesis, promoting tissue repair. In contrast, the control group exhibited less collagen deposition with lower staining intensity. Collagen fibres were distributed unevenly, with lower fibre density and some areas showing irregular fibre alignment, reflecting insufficient collagen synthesis during wound healing. At various time points, collagen deposition lagged behind the inflammatory response in the control group, and angiogenesis occurred more slowly, resulting in a delayed wound healing process. Quantitative analysis of the Masson’s staining revealed that the collagen fibre area ratio in the experimental group was consistently higher than in the control group at every time point, indicating more abundant collagen deposition and a faster healing process (Figures 4F,G).

### 3.7 Immunohistochemical analysis

**FIGURE 5:**
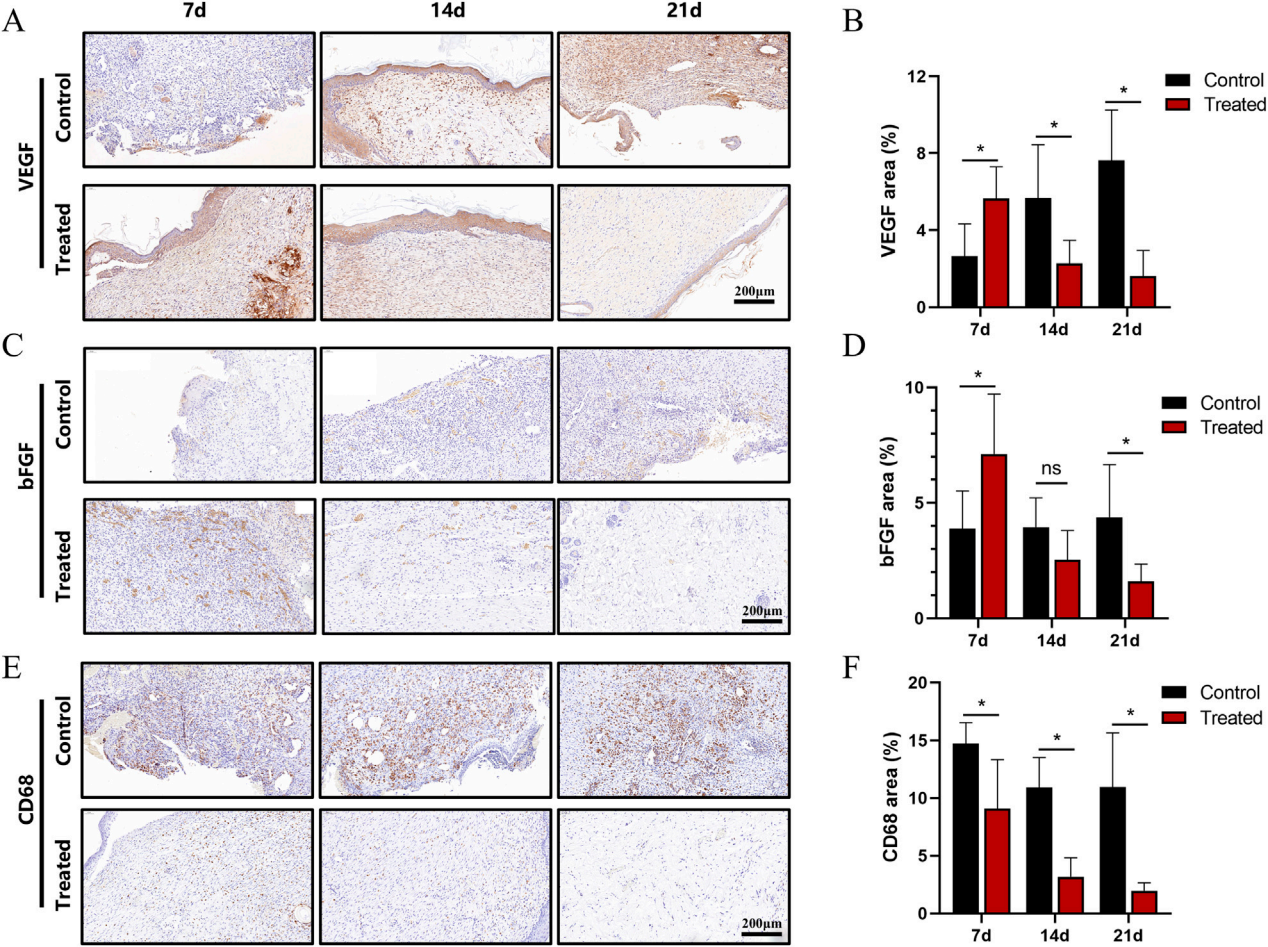
Immunohistochemical analysis of the wound area in a full-thickness skin injury model. **(A)** Immunohistochemical staining of VEGF in wound tissues at days 7, 14, and 21. **(B)** Quantification of VEGF-positive signals. **(C)** Immunohistochemical staining of bFGF at corresponding time points. **(D)** Quantification of bFGF-positive signals. **(E)** Immunohistochemical staining of CD68 at days 7, 14, and 21. **(F)** Quantification of CD68-positive signals. Scale bar: 200 μm. All data are expressed as mean ± SD (*n* ≥ 6). Statistical significance determined by two-tailed t-test; ns indicates no significance, *P < 0.05, **P < 0.01, ***P < 0.001.

Immunohistochemistry was employed to assess wound healing at the protein level. VEGF expression is a marker of neovascularisation. The results showed that the treatment group had significantly higher VEGF expression on day 7 compared to the control group. By day 14, VEGF levels in the treatment group had decreased, while the control group still exhibited relatively high levels of VEGF expression. In the later stages of wound healing, VEGF expression in the control group remained concentrated around blood vessels, whereas only a small amount of positive signal remained in the treatment group, mainly distributed in the basal layer of the epidermis or around hair follicles (Figures 5A,B).

BFGF, a key factor in angiogenesis, can synergistically work with VEGF and enhance fibroblast activity, thereby shortening the healing time. It exhibited strong positive signals in the new granulation tissue and basal layers. The results showed that bFGF expression in the treatment group peaked on day 7, with quantitative analysis indicating higher expression levels than in the control group. By day 14, the expression in the treatment group had largely decreased, while the control group still exhibited moderate levels of expression. On day 21, the expression in the treatment group was almost identical to that in normal tissue. These results suggest that the treatment group had a faster progression of granulation tissue growth and fibre remodelling compared to the control group (Figures 5C,D).

CD68, a marker for mononuclear-macrophages, was used to evaluate the inflammatory response in the wound area. On day 7, the control group showed a large number of positive signals, which gradually decreased over time. In contrast, the treatment group exhibited consistently lower levels of CD68 expression at all time points, with values only slightly higher than those in normal tissue. This indicates that the dressing used in the treatment group had anti-inflammatory effects (Figures 5E,F).

## 4 Discussion

Severe wounds have long been a concern for healthcare professionals, with slow wound healing being one of the primary challenges (Guest et al., 2017). Currently, there is a need for an effective wound dressing that can accelerate the healing process, is cost-effective, and easy to manufacture (Sasan et al., 2024). In this study, we developed a bilayer electrospun fibre scaffold dressing, which was demonstrated through a series of *in vitro* and *in vivo* experiments to have great potential as a wound dressing. In this study, we developed a bilayer electrospun fibre dressing composed of TPU, CS and ZnO. This design integrates cost-effective production with simplified fabrication processes while demonstrating favorable functional performance. Through systematic *in vitro* and *in vivo* assessments focused on biological responses—including cytocompatibility, antibacterial efficacy, and pro-healing activities—we validate the therapeutic potential of this scaffold for wound management applications. The current work prioritizes biological verification of the electrospun system, with mechanistic refinements to be explored in subsequent investigations.

First, regarding the physicochemical properties of the material, SEM and EDS results confirmed that the fabricated fibre scaffolds exhibited excellent morphological uniformity and a homogeneous distribution of ZnO particles. The stable fibre diameter and low coefficient of variation suggest that the electrospinning process was stable and that TPU and CS were well compatible. The introduction of ZnO nanoparticles did not significantly alter the fibre morphology but did impact the mechanical properties, resulting in a decrease in tensile strength and elongation at break. This finding is consistent with prior research outcomes (Alshabanah et al., 2021). However, the bilayer scaffold design effectively compensated for this drawback, maintaining good mechanical performance while preserving the biological activity of the functional materials (Touré et al., 2020). Research has established that cellular adhesion on nanobiomaterials exhibits a positive correlation with surface hydrophilicity. Lower water contact angles indicate enhanced hydrophilic properties, which facilitate cell adhesion and proliferation. For electrospun fibrous scaffolds, surface hydrophilicity is governed by fibre composition, morphology, diameter, and alignment.

TPU, being a hydrophobic material, does not naturally conform well to wound surfaces, which limits its application in wound healing due to poor contact with the wound (Patil et al., 2022). To address this issue, chitosan was incorporated into the scaffold, significantly lowering the water contact angle of the fibre scaffold (Kong et al., 2010). The inclusion of chitosan also enhanced the biological effects of the scaffold (Chen et al., 2025) which significantly accelerates the healing kinetics (Li et al., 2025). As a wound dressing, the material should protect the wound from external damage; however, excessive hydrophilicity could lead to water droplets entering the wound and interfering with the healing process (Oliveira et al., 2014). To mitigate this, we fabricated a layer of TPU nanofibres on the surface of the TPU/CS@ZnO scaffold using a continuous electrospinning process, endowing the dressing with some waterproof properties. The therapeutic advantages of hydrophobic-hydrophilic bilayer configurations in wound management have been demonstrated in multiple studies (Miguel et al., 2019; Miguel et al., 2017). Evidence-based data indicate an optimal WVTR of 2,000–2,500 g·m^−2^·d^−1^ for advanced wound dressings. Maintaining this target WVTR range prevents exudate accumulation while effectively protecting nascent granulation tissue and adjacent healthy tissue from maceration (Figueira et al., 2016). The hydrophobic TPU layer and hydrophilic TPU/CS@ZnO layer together created a hierarchical microenvironment conducive to cell attachment, migration, and moisture balance maintenance at the wound site. The overall good breathability of the scaffold was beneficial for promoting oxygen exchange and metabolism in the wound area, providing the necessary physiological conditions for wound repair (Kong et al., 2023).

Zinc is an essential trace element for the human body, and an appropriate amount of Zn can promote wound healing (Sasan et al., 2024). However, the concentration-dependent biological effects of ZnO have been well-established in multiple studies. High doses of Zn can be toxic to cells (Wang et al., 2024). Through *in vitro* cytotoxicity tests, we verified the effects of different Zn concentrations on cell proliferation. The results showed that the biological effects of Zn are dose-dependent, and we optimized the ideal ZnO loading amount based on these findings. The 1.0% ZnO concentration selected in this study can be considered as the most suitable concentration, balancing biological activity and safety.

Biocompatibility is the cornerstone for the clinical translation of wound dressings. In this study, hemolysis tests and L929 cell culture experiments confirmed that the scaffold has good compatibility with both blood and tissue. The hemolysis rate of the scaffold extracts was below 5%, meeting the ISO 10993-4 biological material safety standards. Live/dead cell staining showed that cells maintained a normal spindle shape on the scaffold surface, with very few dead cells, further confirming its low toxicity and excellent cell compatibility. In terms of promoting cell proliferation and migration, the ZnO-loaded scaffold exhibited better biological effects compared to non-loaded groups in both CCK-8 and scratch assays. The Transwell experiment further validated that the introduction of ZnO significantly enhanced cell migration ability. Some studies suggest that Zn^2+^ ions may regulate signalling pathways such as PI3K/AKT or MAPK, thereby enhancing cell migration and tissue remodelling at the wound site (Zhang et al., 2025).

The results of animal experiments further confirmed the tissue repair effects of the scaffold in a full-thickness skin wound model. The treatment group showed faster wound closure and better tissue reconstruction at all time points, with nearly complete healing by day 14. An ideal wound dressing should interact therapeutically with the wound bed while exhibiting non-adherent properties to minimize secondary trauma during dressing changes and prevent patient discomfort (Palo et al., 2019). In experimental observations, dressings applied to treated wounds could be removed effortlessly without disrupting newly formed tissue. In contrast to the traditional dressings used in the control group, which often adhered to granulation tissue and caused bleeding during dressing changes, the non-adhesive feature of the nanofibre scaffold effectively protected the newly formed tissue and significantly reduced secondary injury (Marques et al., 2023). Photographic documentation of wound gross morphology revealed the presence of reticular neogranulation tissue, a pattern associated with impaired angiogenesis and delayed healing. This aberrant tissue architecture unequivocally compromises wound repair processes. This demonstrates a clear clinical advantage over traditional dressings.

Histological (H&E and Masson) staining analysis further revealed the mechanisms underlying the scaffold’s promotion of wound healing at the cellular and tissue levels. The treatment group formed dense granulation tissue early in the healing process, with abundant and orderly collagen fibre deposition in the dermis, indicating a positive role in extracellular matrix remodelling. Quantitative analysis of Masson’s staining showed that the collagen fibre deposition in the treatment group was significantly higher at all time points compared to the control group, suggesting its key role in promoting connective tissue formation and structural reconstruction. This healing trajectory aligns with established wound progression patterns documented in prior murine model studies (Yu et al., 2019).

Immunohistochemical analysis revealed the regulatory mechanisms of the dressing on molecular pathways related to wound healing. The temporal expression patterns of VEGF and bFGF indicated that the treatment group rapidly triggered angiogenesis in the early stages of healing, and subsequently reduced the expression in the later stages, preventing excessive neovascularisation and scar formation (Frank et al., 1995). The influence of the immune microenvironment on wound healing rates has been well documented (Bai et al., 2020). Acute inflammation is necessary for initiating wound repair; however, its prolonged presence can lead to tissue damage, cellular dysfunction, and delayed healing (Khan et al., 2021). The low expression of CD68 in the treatment group suggests that the scaffold possesses anti-inflammatory properties, through the reduction of macrophage recruitment and inflammatory cytokine secretion, thus creating a favourable microenvironment for repair and accelerating the healing process (Le et al., 2022).

As a nanomaterial with anti-inflammatory activity and regenerative potential, ZnO NPs played multiple roles in regulating the wound microenvironment. First, Zn^2+^, an important trace element, promoted cell migration and proliferation and enhanced angiogenesis (Lin et al., 2017). Studies have shown that Zn^2+^ stimulates fibroblasts to express matrix metalloproteinase (MMPs) and growth factors, promoting basement membrane reconstruction and extracellular matrix remodelling (Yadav et al., 2022). It also exerts a chemotactic effect on endothelial cells, promoting angiogenesis (Li et al., 2024), which is consistent with the high expression of VEGF and bFGF observed in the treatment group on day 7.

Second, ZnO NPs demonstrated an advantage in modulating immune cell activity. Previous studies have shown that ZnO NPs can inhibit pro-inflammatory cytokines such as TNF-α and IL-1β through the regulation of NF-κB and MAPK pathways, while promoting the release of anti-inflammatory cytokines such as IL-10 (Wang et al., 2022). In our experimental results, this regulatory effect manifested as reduced macrophage infiltration and a quicker resolution of inflammation in the treatment group, leading to a faster transition to the proliferative phase of tissue repair.

Furthermore, the size effect of ZnO NPs contributed to their sustained release behaviour in the scaffold (Augustine et al., 2014), maintaining a low concentration of Zn^2+^ for stable, prolonged release. This “low-dose, long-term regulation” mechanism provided support throughout multiple stages of wound healing, facilitating a smoother, continuous regenerative process.

Composite materials such as chitosan and hyaluronic acid exploit hydrolyzable bonds to establish pH-responsive systems, which modulate therapeutic release intensity according to physiological microenvironmental changes. This strategy effectively mitigates the burst release limitations inherent to electrospun fibre-based drug delivery (Tian et al., 2025). However, TPU-based scaffolds maintain structural integrity across wound pH gradients—a trait advantageous for long-term mechanical support but limiting for adaptive drug release.

## 5 Conclusion

This study successfully developed an electrospun nanofibrous scaffold based on a polyurethane/chitosan hybrid system and enhanced its functionality through the uniform loading of ZnO NPs. Experimental results demonstrated that the TPU/CS@ZnO scaffold exhibits excellent material properties, including mechanical performance, hydrophilicity, and breathability, which meet the basic requirements for wound dressing applications. *In vitro* cell culture experiments and hemolysis tests confirmed that the scaffold is highly compatible with both blood and cells. The introduction of low concentrations of ZnO did not induce cytotoxicity; instead, it displayed a positive effect on promoting fibroblast proliferation and migration, providing an ideal microenvironment for wound repair.

The ZnO nanoparticles exerted multiple effects by slowly releasing Zn^2+^ ions, which played a key role in reducing local inflammation, balancing the immune microenvironment, promoting angiogenesis, and enhancing collagen deposition, thus accelerating the wound healing process. The treatment group showed significant advantages over the control group in terms of new tissue formation, angiogenesis, and the clearance of inflammatory cells, offering strong theoretical support for the design of multifunctional wound dressings.

Overall, the comprehensive functionality of this scaffold indicates its promising potential for future clinical wound dressing and tissue engineering applications.

## 6 Limitations

Like previous studies, our research has some limitations that could be addressed in future investigations. First, this study employed a rat full-thickness skin wound model to verify the scaffold’s effect on wound healing; however, biological differences between species may affect the applicability of the results to clinical settings. Second, the specific molecular mechanisms and signaling pathways involved remain relatively underexplored, and more in-depth mechanistic analyses are needed in the future. Last, this study mainly focused on the preliminary investigation of the scaffold’s effect on wound healing rates, and future research should introduce commercially available dressings and set appropriate controls for comparison.

## Data Availability

The raw data supporting the conclusions of this article will be made available by the authors, without undue reservation.
